# Dual Energy X-ray Absorptiometry Scanning and Bone Health: The Pressing Need to Raise Awareness Amongst Pakistani Women

**DOI:** 10.7759/cureus.5724

**Published:** 2019-09-22

**Authors:** Farah Anwar, Hiba Iftekhar, Tasneem Taher, Syeda K Kazmi, Fatima Z Rehman, Minhal Humayun, Samar Mahmood

**Affiliations:** 1 Internal Medicine, Dow University of Health Sciences, Karachi, PAK; 2 Internal Medicine, Dow University of Health Sciences, Karachi , PAK

**Keywords:** dual energy x-ray absorptiometry (dexa), bone health, knowledge, attitudes, practices, pakistan, bone scan, public health, osteoporosis, females

## Abstract

Introduction

The use of dual-energy x-ray absorptiometry (DEXA) scanning is instrumental in better management of osteoporosis. This study aimed to assess the level of knowledge about DEXA scanning and bone health in the women of Karachi, as well as to analyze their practices concerning the scan and increase their knowledge and awareness regarding the same.

Methodology

The sample size for this cross-sectional study was 384. Data were collected using a self-devised and validated questionnaire, consisting of four sections: social demographics, general knowledge about DEXA scanning, practices regarding DEXA, and knowledge about bone health. The data were analyzed using Statistical Package for the Social Sciences (SPSS) version 23 and associations between multiple variables calculated, using independent sample t-test and Pearson’s chi-squared test.

Results

Only one-third of our sample population had heard about DEXA scanning and amongst them, nobody had complete knowledge about it. The mean score of general knowledge of DEXA (5.3±2.0) was higher than that of knowledge about the conditions in which DEXA scanning is recommended (2.7±2.1). The knowledge score showed a significant correlation with education (p=0.007) and employment (p=0.001). Only 7.6% of the sample population had taken a DEXA scan and knowledge and employment status were found to have significant associations with practices (p value=0.000 and 0.001, respectively).

Conclusions

The awareness levels regarding DEXA scans and bone health should be evaluated amongst similar and other groups of people and effective measures be put into application to educate the public and to guide them towards better prevention and management of osteoporosis.

## Introduction

Dual-energy x-ray absorptiometry (DEXA) scan is a quick and accurate test that is widely used for measuring bone mineral density (BMD) and to diagnose osteoporosis. Osteoporosis affects more than 200 million people worldwide and one-in-three women above 50 years old [1-2]. The National Institute of Health defines osteoporosis or ‘porous bone,’ as a systemic skeletal disease characterized by low bone mass and structural deterioration of bone tissue which leads to an increased risk of fractures of the wrist, spine, and hip [2]. The rate of fractures resulting from osteoporosis globally has been recorded at about 8.9 million per year which intensifies the economic burden that results from the pain, restricted mobility, and impaired functionality of the individual [1]. It is four times more common in women than in men, particularly in the postmenopausal phase since estrogen production falls [3]. Therefore, DEXA screens patients with low BMD, so as to give them proper prophylactic treatment to avoid fractures and decrease the risk of morbidity.

An advantage of DEXA is that it is a noninvasive scan with low radiation exposure. The World Health Organization (WHO) recognizes DEXA as the gold standard to assess bone density [2]. Most common examination sites for DEXA are the hip and lower spine. It is reported that 20-30% of patients that have had a hip fracture die within a year, which highlights the cruciality of fractures at this site [4]. Pakistan has more vitamin D deficiency than USA, which is one of the prime factors that causes lower bone density. Also, there are lesser DEXA machines available in Pakistan to screen patients, coupled with an extremely low rate of the prevalent awareness regarding the importance of the same [5]. It is estimated that osteoporosis cases will rise to more than seven million in Pakistan by 2020 [1]. A total of 13-18% of women above 50 years of age in USA are osteoporotic and in Pakistan, the figure is 13%, although Pakistan is a smaller country with just 60% the population of USA [6]. Numerous studies have been conducted that have assessed the knowledge of osteoporosis prevention, particularly amongst women and the majority of the sample population has been found to be informed regarding the risk factors of the disease [7]. In contrast, there has been insufficient research done in Pakistan about women’s knowledge regarding DEXA scanning and osteoporosis and with this research, we aimed to fill that void and direct future work on the same theme.

The purpose of this study was to assess the knowledge, attitude, and practices towards DEXA scanning and bone care among women above the age of 30 years, in Pakistan’s most populous city Karachi. Our secondary objective was to increase basic awareness regarding the knowledge and availability of DEXA in our target population and to make them realize the importance of having it done via an informative supplement given to them after they filled out the questionnaire. Those with low BMD were encouraged to get a treatment plan involving exercise, an adequate intake of calcium and vitamin D, and cessation of smoking amongst other required changes of lifestyle.

## Materials and methods

This cross-sectional study was conducted in the city of Karachi within a span of nine weeks amongst women aged 30 years and above within and outside the hospitals, at homes and across different working environments like schools, offices, and public places like shopping malls, theaters, and markets widely distributed across the city. Participants were recruited through convenience sampling, filtered based on their understanding of English, and level of education where individuals without a secondary level education or who did not understand English were excluded. The inclusion criteria were women aged 30 years and above. The sample size was found to be 384 with a confidence interval of 95% along with a 5% margin for error. The sample was calculated by an online statistical software provided by the website, ‘openepi.com.’

The participants were interviewed between the months of August and October 2018. Multiple methods were devised to avoid interviewer bias, including manual interviews being conducted by a team of similarly trained individuals-in language, phrasing of questions, manner of interviewing, and attire. A total of 426 women were approached out of which 384, the aimed sample size, responded. Thus, the compliance rate was found to be 90%. Written informed consent was taken and participants were assured that all data would be kept confidential. The Institutional Review Board (IRB) waived the need for approval for this study since it was a basic survey conducted amongst the general population. The Declaration of Helsinki was abided by.

A self-devised questionnaire was structured based on literature search and findings discussed in previous articles, consisting of a set of 25 questions and comprising of four sections and was reviewed by two doctors to confirm validity. A pilot study consisting of 40 participants was conducted initially. The first eight questions dealt with sociodemographic details, in which the participants were asked to provide information regarding their age, occupation, monthly income, number of pregnancies, and children breastfed. The next section contained 11 general knowledge questions on DEXA scan, which tested the recipients’ knowledge on the scan’s use, its investigative conditions, precautions required, procedure type, the recommended age for a DEXA scan, conditions under which the scan should be taken, contraindications, and how often the scan should be performed under both normal and/or low BMD [8]. Poor, moderate, and good scores on general knowledge of DEXA were determined as the number answered out of the total of 11 questions on it, less than or equal to 2 being poor, 3-5 moderate, 6-8 good, and 9-11 marked as an excellent score. The score for conditions under which DEXA is undertaken was determined from the conditions correctly identified out of a total of 10 correct options listed in the question on it, less than 2 being poor, 3-5 moderate, 6-8 good, and 9-10 marked as an excellent score. The third section questioned women whether they had taken the scan and their reasons for taking it. The fourth section aimed at surveying the participants’ attitudes regarding bone health. The concluding question comprised of a series of true and false statements to test knowledge of overall bone health and osteoporosis management. A brief overview of facts related to DEXA and bone health was presented to the recipients for their knowledge after they completed the interviews.

Data were analyzed and verified through Statistical Package for the Social Sciences (SPSS) version 23.0 for Windows. The entire sample was approved for final analysis as missing data did not exceed more than 5% and there was no imputation. Means, frequencies, percentages and standard deviations of certain variables were calculated and the association between multiple variables was found using independent t-test and Pearson’s chi-squared test.

## Results

The average age of the sample population (N=384) was (44.5 ± 8.1) years with the minimum age being 30 (n=14, 3.6%) and maximum age being 79 (n=1, 0.3%) years. Majority of the population had a bachelor’s degree (n=183, 47.7%) with matric/O level being the lowest level of education (n=20, 5.2%). Most individuals of the study population were unemployed (n=223, 58.1%) but about half belonged to high socioeconomic background. Those employed in the medical field constituted only 6.5% (n=25) of the study population. Among the participants (N=384), nobody had complete knowledge about DEXA scanning. Only one-third (n=128) of the population answered ‘Yes’ to the question ‘Have you ever heard of DEXA scanning?’ Maximum overall knowledge recorded was 91% (10 out of the 11 questions on general knowledge of DEXA answered correctly) but this was observed with only two out of the 128 who knew about DEXA. A meager percentage of the population (n=28, 21.9%) could answer only six questions correctly whereas two people could not answer any of those 11 questions on general knowledge regarding DEXA. The mean score of general knowledge of DEXA (5.3±2.0) was higher than the mean score of knowledge about the conditions in which DEXA scanning is recommended (2.7±2.1), where the maximum number of questions answered correctly was 10 [Table 1]. A significant number of the population (n=31, 24.2%) had knowledge about one of the conditions. One person knew about all the conditions whereas 12 people had no knowledge about the conditions under which the scan is recommended. Out of the entire sample population, only 21.9% (n=84) knew that DEXA is strongly recommended for women older than 65 years. Additionally, knowledge of the health field professionals about conditions that have a strong implication for DEXA was surprisingly poor especially for the condition ‘history of gastrointestinal disorder’ only three out of 25 had knowledge. Similarly, ‘history of rheumatoid arthritis’ and ‘history of smoking’, were both positively acknowledged by only seven and the most disappointing figure of only two medical personnel giving the right answer for the correct age for DEXA in patients without any risk factors.

**Table 1:** Descriptive statistics of study participants SD: Standard deviation

					N=384 (100%)		
Sociodemographics							
Mean age ± SD					44.5 ± 8.1		
Education	Matric/ O level				20 (5.2)		
	Intermediate / A level				37 (9.6)			
	Bachelors				183 (47.7)			
	Post graduate				144 (37.5)			
Employment status	Unemployed				223 (58.1)		
	Employed				91 (23.7)			
	Self employed				45 (11.7)			
	Employed in medical /health field.				25 (6.5)			
Family Income	10 to 50K				51 (13.3)			
	50 to 100K				135 (35.2)			
	Above 100K				198 (51.6)			
Knowledge	People who answered ‘Yes’ to the question ‘’Have you ever heard of DEXA scanning?’’				128 (33.3)		
	Mean score of general knowledge about DEXA scanning ^a^				(5.3±2.0)			
	Mean score of knowledge about conditions^a^	(2.7±2.1)			
Questions answered	General knowledge on DEXA				
0-2	Poor		12 (9.4)			
3-5	Moderate		54 (42.2)			
6-8	Good		56 (43.8)			
9-11	Excellent		6 (4.7)			
Questions answered	Knowledge about conditions						
0-2	Poor			72 (56.3)			
3-5	Moderate			43 (33.6)			
6-8	Good			10 (7.8)			
9-10	Excellent			3 (2.4)			

Table 2 highlights the knowledge of the participants about bone health, assessed for the entire sample population (N=384). Only three out of the 384 people had 100% knowledge about bone health whereas 13 participants had absolutely no knowledge regarding bone health (not shown in the table). Four-fifths of the population had knowledge about the facts that the most important time to build bone strength is between 9 to 17 years of age, and that walking has a great effect on bone health. However, only 53 (13.8%) individuals of the study population knew that lower weight women are more prone to suffer from osteoporosis, which was observed to be the least well-known fact.

**Table 2:** Knowledge regarding bone health ^a ^These are false statements and the table shows the number of participants who marked it as false in the questionnaire.

Statement	Frequency of people who had the right knowledge (%)	
High impact exercise (weight training) improves bone health.		208 (54.2)
Most people gain bone mass after 30 years of age.^a^		185 (48.2)
Lower weight women have osteoporosis more than heavier women.		53 (13.8)
The most important time to build bone strength is between 9 to 17 years of age.		320 (83.3)
Normally bone loss speeds up after menopause.		305 (79.4)
High caffeine combined with low calcium intake increases the risk for osteoporosis.		207 (53.9)
There are ways to manage and treat osteoporosis after it develops.		277 (72.1)
Walking has a great effect on bone health.		314 (81.8)
Osteoporosis affects both men and women.		247 (64.3)
After menopause, women not on estrogen need about 1500mg of calcium (e.g. 5 glasses of milk) daily.		139 (36.2)
Replacing hormones after menopause cannot slow down bone loss.^a^		118 (30.7)

Table 3 highlights the percentage of the participants who had taken a DEXA scan along with those who had never taken a DEXA scan, their reasons for undergoing the scan and the barriers that prevented them, respectively. Only 7.6% of the sample population had taken a DEXA scan, out of whom 62.1% were those who were prescribed the scan by a doctor. ‘Previous history of fractures/low BMD’ (n=5, 17.2%), ‘suggestions by friends/family members’ (n=3, 10.3%), and ‘to create a workout plan’ (n=1, 3.4%) were less commonly reported reasons for opting for the scan. The minimum and maximum numbers of times the scan was taken by the participants was one and three, respectively. Most participants (n= 21, 72.4%) had gone for the scan only once and only three participants (10.3%) had undertaken the scan thrice. Out of the 92.4% (n=355) who had never taken a DEXA scan, the most common barrier was ‘lack of awareness’ as reported by 70.1% (n=249) of the participants, whereas ‘fear’ (n=2, 0.2%) and ‘accessibility issues’ (n=3, 0.8%) were less commonly reported. ‘Lack of a doctor’s recommendation’ (n=90, 25.4%) was another reason given by one-fourths of this group of participants.

**Table 3:** Practices regarding dual-energy x-ray absorptiometry scan ^a^ All percentages were calculated out of the 29 people who had taken DEXA. ^b^ All percentages were calculated out of the 355 people who had not taken DEXA.

	n (%)
People who had taken DEXA	29 (7.6)
Reasons for taking DEXA (n=29)^a^	
Doctor prescribed it	18 (62.1)
Friend/ family member suggested it	3 (10.3)
Had a history of fractures and wanted to know BMD	5 (17.2)
Wanted to assess the total body fat and create a dietary/workout plan	1 (3.4)
Others	2 (6.9)
People who had not taken a DEXA scan	355 (92.4)
Barriers preventing them from taking a DEXA scan (n=355)^b^	
Lack of awareness	249 (70.1)
Cost of the procedure	12 (3.4)
Weren’t recommended by doctor	90 (25.4)
Accessibility issues	3 (0.8)
Fear	2 (0.6)

Table 4 indicates that the knowledge score showed a statistically significant correlation (p-value <0.050) with education (p=0.007) and employment (p=0.001). However, a significant association between knowledge score and mean age (p=0.061) could not be reached.

**Table 4:** : Association between socio-demographic characteristics and knowledge about dual-energy x-ray absorptiometry scanning

Variable	Knowledge		P value
	Yes n=128 (33.3%)	No n=256 (66.7%)	
Mean age ±SD	43.7 ±8.7	44.8 ±7.8	0.241
Education			0.007
Matric/ O levels	2 (1.6)	18 (7.0)	
Intermediate/ A levels	7 (5.5)	30 (11.7)	
Bachelors	60 (46.9)	123 (48.0)	
Postgraduate	59 (46.1)	85 (33.2)	
Employment			0.000
Unemployed	56 (43.8)	167 (65.2)	
Employed	42 (32.8)	49 (19.1)	
Self-employed	11 (8.6)	34 (13.3)	
Employed in Medical field	19 (14.8)	6 (2.3)	

Table 5 shows the impact of knowledge and demographic characteristics on undertaking a DEXA scan. Knowledge and employment status both had a statistically significant association with practice (p value= 0.000 and 0.001) respectively. Approximately half of the people who had undergone DEXA scanning fell in the highest salary range. However, the relationship between practice and income was not statistically significant (p value=0.881). The maximum number of children breastfed were eight and of these women, three-fourths had not taken the scan. Mean age (p=0.061) and education (p=0.255) had no statistically significant association with practice. In the sample population, 17 women (4.42%) were aged 60 years or above, but only five of them opted to undergo DEXA scanning.

**Table 5:** Association of knowledge and sociodemographic characteristics with practice of participants

Variable	Practice		P value
	Yes n=29 (7.6 %)	No n=355 (92.4%)	
Mean age ±SD	47.9 ± 10.0	44.2±7.9	0.061
Education			0.255
Matric/O level	2 (6.9)	18 (5.1)	
Intermediate/A level	0 (0.0)	37 (10.4)	
Bachelors	17 (58.6)	166 (46.8)	
Post graduate	10 (34.5)	134 (37.7)	
Employment status			0.001
Unemployed	12 (41.4)	211 (59.4)	
Employed	6 (20.7)	85 (23.9)	
Self employed	4 (13.8)	41 (11.5)	
Employed in medical field	7 (24.1)	18 (5.1)	
Income			0.881
10-50K	3 (10.3)	47 (13.2)	
50-100K	12 (41.4)	123 (34.6)	
Above 100K	14 (48.3)	185 (52.1)	
Knowledge			0.000
Yes	27 (93.1)	101 (28.5)	
No	2 (6.9)	254 (71.5)	
Number of children breastfed			0.311
Minimum number (0)	3 (10.3)	37 (10.4)	
Maximum number (8)	1 (3.4)	3 (0.8)	

**Figure 1:**
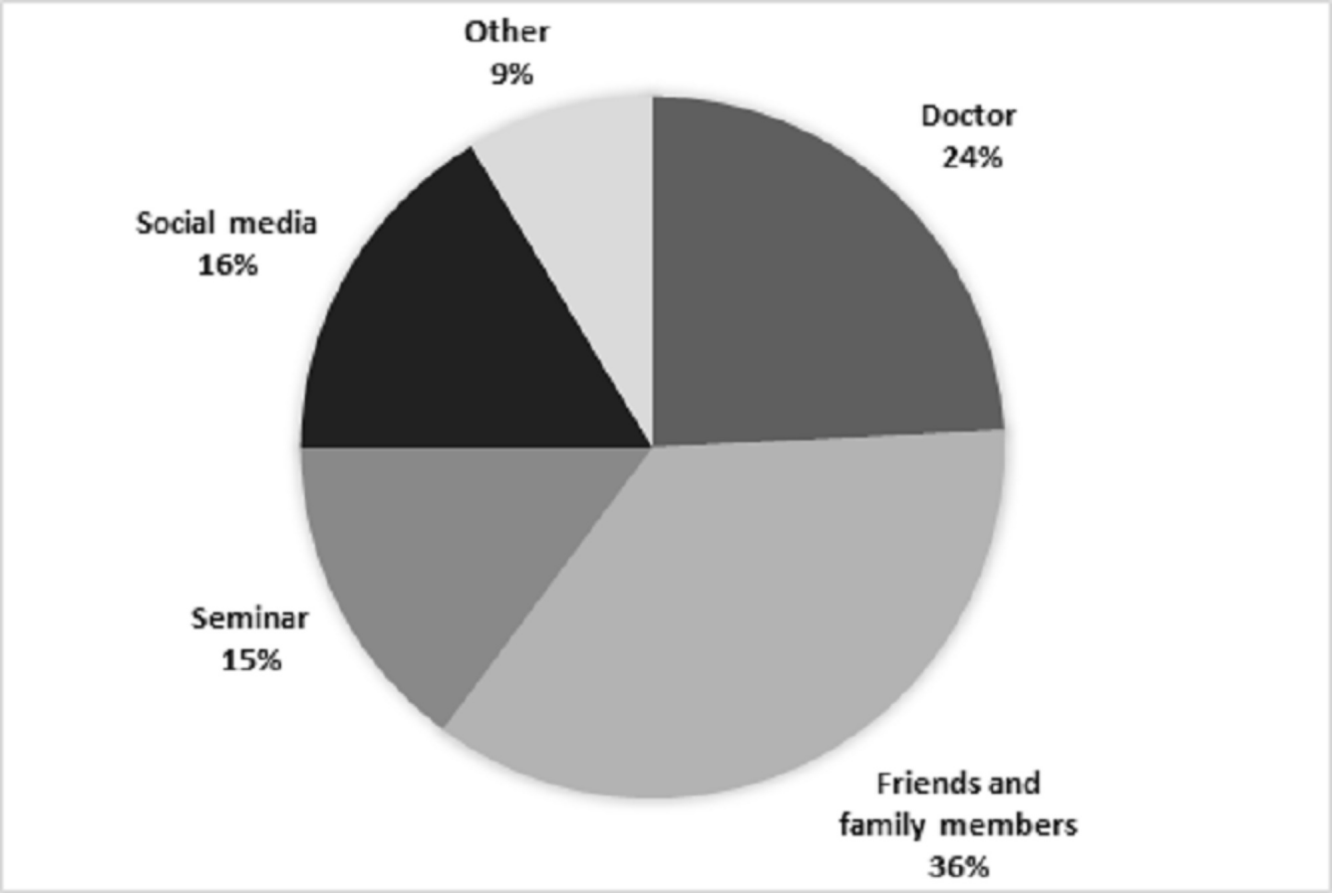
Sources of knowledge regarding dual-energy x-ray absorptiometry scan

Figure 1 illustrates the sources of knowledge about DEXA scan for the 128 participants who had heard about DEXA. The most common source of knowledge was ‘friends and family members’ (n=46, 36%). Doctors were identified as the second most common source (n=31, 24%), while ‘seminars’ played a role in educating only 15% (n=19) of the population. Some other knowledge sources e.g. Wikipedia and MBBS course books were reported by only 8.6% (n=11) of the participants.

## Discussion

To the best of our knowledge, this investigation was the first study that estimated the knowledge and practices regarding DEXA scanning in our country. Previous research found the BMD of Pakistani women to be lower than that of European women, and another study asserted that the prevalence of osteoporosis is generally higher in Asians [9-10]. These facts consequently highlight the significance of evaluating our public’s status of knowledge regarding bone health and DEXA scanning, as well as the dire need to educate the masses about the latter - both of which this study aimed to do.

As expected, our results were discouraging, with only one-third of the participants having prior knowledge about DEXA. Among them, respondents scored better on the section relating to general knowledge about DEXA, with 86% showing moderate-to-good level of knowledge which they had acquired through friends and family, primarily, and doctors, secondarily. Social media and seminars played a surprisingly poor role in educating people even though in this era, these are considered to be the most efficient mediums of spreading information. In addition, comparable with scientific literature from the US, the majority amongst those who knew about DEXA recognized it as the gold standard to diagnose low BMD [11]. However, the question that most people, including the doctors of the sample population, did not answer correctly was the age at which a DEXA scan is recommended in people not having any risk factors for bone loss; it was most commonly thought to be ‘30 and above’ as opposed to ‘65 and above’ that is mentioned in recommendation guidelines [4]. This can be attributed to the fact that our sample population comprised explicitly of women aged 30 years or older, so the participants could have concluded that to be the age when the scan is recommended as well. However, this age group was chosen to be the sample population in our study bearing in mind earlier research conducted in Karachi, which had concluded BMD to be decreased in 54% of women older than 30 years [12]. Therefore, it was thought to be imperative to increase awareness about DEXA scanning among women who fell in this age bracket.

The knowledge about conditions requiring the need to undergo the scan was not found to be satisfactory either, with more than half of the sample population (56.3%) scoring poorly. This ties in with literature from Thailand that has mentioned a lack of awareness about the different conditions that increase the risk for secondary osteoporosis, and are, therefore, conditions that require BMD testing through DEXA [13]. These include age above 65 years, menopause, history of fall, history of gastrointestinal disorders, alcohol consumption, glucocorticoid use, and rheumatoid arthritis because all of them pertain to an increased risk of bone deterioration. Of these, respondents of our study were aware of menopause, which corresponds to scientific literature that has mentioned the correlation of menopause and osteoporosis many times over. A decline in the quantities of hormone like estrogen after menopause increases the odds of suffering from osteoporosis for postmenopausal females, by accelerating the osteoclastic activity of bone remodeling [14]. However, as per recommendations by the US Preventive Services Task Force, the condition that poses a more pressing need for the scan is being 65 years or older, but only 21.9% of our study population had this knowledge [4]. The reason for this lack of awareness can be linked to the paucity of diagnostic facilities like DEXA and of national registries and published data in Pakistan, as mentioned by Khan et al. in their study [5]. Surprisingly, even in the USA that has the highest number of DEXA machines, the number of people getting scanned is declining for reasons that are being studied [15-16].

Furthermore, a substantial number of our participants knew about the crucial age for bone building, the positive effect of walking on bone health, and the negative impact of menopause on bone loss. All of these factors have long been discussed in great detail across the literature. However, even though more than three-quarters knew that bone loss speeds up after menopause, less than half thought it to be important to follow-up with a daily intake of 1500 g of calcium. This highlights the disregard for adequate calcium consumption by the women of Karachi, which is an important modifiable factor for osteoporosis. This is also in accordance with a study carried out in Khyber Pakhtunkhwa, Pakistan, that showed dietary calcium intake by postmenopausal women in that community to be far less than WHO recommendation [17]. In addition, even women in western countries like USA, Germany, and Ireland were found to not have been consuming calcium as recommended by WHO either [18]. Also, more than four-fifths of the population were unaware of lower-weight women being more prone to osteoporosis, which further cements the idea that people do not give due regard to dietary modifications for prevention of diseases that do not present overtly. In contrast, an encouraging finding of our study was that 31% of our respondents showed a positive response to awareness about Hormone Replacement Therapy (HRT), which was at variance with an earlier study conducted in Karachi, in which only about two percent of the respondents had known about it [19].

The practices regarding DEXA scanning were especially disappointing since only 7.6% of the sample population reported as having taken the scan previously. Moreover, a substantial number of these people had undergone the procedure only upon recommendation by a doctor. This parallels literature from an Ontarian study which estimated that 80% of the BMD tests carried out were on physicians’ orders [20]. Coupling this fact with the poor knowledge score observed even amongst the doctors of the study population, regarding the conditions in which DEXA is recommended, there rises a dire need to spread awareness among both sections of people alike. Moreover, considering that nearly all those who opted for the scan were a part of the group of respondents that had prior knowledge of DEXA and that the main barrier to practice was found to be ‘lack of awareness’ as reported by 70% of the participants, it is highly plausible that increasing awareness would result in an improvement of practice of DEXA examination. In addition, the lack of interest shown by people in getting their BMD assessed suggests that painless and symptomless conditions like osteopenia are not of foremost concern to people, even though osteoporotic fractures are associated with high mortality [21]. Furthermore, the importance of using DEXA to screen for osteoporosis, especially in the elderly, was made apparent from a cohort study in USA that demonstrated a reduced incidence of fracture among those senior citizens who got their BMD tested as a preventive measure [22]. Therefore, it is imperative to provide an impetus to people to get their BMD tested on time.

In addition to that, our results were not in line with the general trend of women of older age being more likely to have undergone BMD testing in developed countries since we observed only a small minority of women over the age of 60 years as having taken the scan [20,23]. However, a similar tendency of younger women having better screening practice than older women in Karachi was previously observed in a study carried out in 2018, which concluded that the most frequent age of diagnosis for breast cancer was in the 40s [24]. No significant association was detected between multiple pregnancies and better DEXA practice either, even though the frequency of osteoporosis in a study was found to be significantly higher in women with five to nine pregnancies compared with women having <5 pregnancies [25]. However, the link between parity and BMD and duration of breastfeeding and BMD remains largely unclear, as some reports have shown that bone loss associated with these factors is recovered after weaning [26].

Although our respondents showed a better grasp on some of the facts related to bone health, the results of this study still highlight an overall insufficient understanding of our community regarding the importance of maintaining good bone health, because a higher knowledge score in this section did not correlate with better practice. This ties in with a research carried out in the US that reported most postmenopausal women describing osteoporosis preventive care as being important but still only half of that sample population had undergone BMD [27]. In Pakistan, many advertisements about dairy products, especially targeting women, are centered around increasing awareness about bone health and lifestyle modification through diet and exercise. However, as Suarez-Almazor ME mentioned in his paper, so far, media campaigns with respect to bone health have not encompassed messages to adhere to recommendation and therapy which can explain the trend in our result that while advertisements increase awareness, they do not necessarily push people towards better screening practice [28]. Also, as indicated by our results, our community was unaware of DEXA being a safe and quick technique; belief in the contrary and associated myths may also prove to be reasons why people deny undergoing this procedure [29]. Additionally, some patients fear being positively diagnosed for a disease, which might also elucidate the low prevalence of BMD testing in this study population.

The limitations of this research include the cross-sectional nature of the study, short duration, and a small sample population size that was neither representative of all the socioeconomic divisions of the society, nor did it encompass the male population. Despite being based on a thorough literature review, our questionnaire was a nonstandard instrument which may limit the comparability of our results with any prior or future studies that use a more standard instrument. Moreover, BMD score of those participants who underwent the scan was not enquired into. Since low BMD can significantly alter the patients’ quality of life, it is important to acquire this data and subsequently assess which age group in our region suffers most from this condition, which is essential in evaluating the economic burden of disease and its sociological importance [30]. However, with this article, we hope to fill the void of research on the existing theme from the country and publish a model that can be improved and replicated on a larger scale to raise more awareness.

## Conclusions

The percentage of population who had prior knowledge of DEXA was extremely low, with an even smaller proportion of individuals reporting to have taken the scan previously. Moreover, considering the lack of knowledge and poor attitude observed among the aimed sample population of a higher socioeconomic background for this study, future studies could use this as a model and extend the survey into the uneducated and underprivileged society, who are expected to have a higher prevalence of low BMD owing to nutritional deficiencies. There is also a growing need to improve the availability of DEXA machines, in addition to bringing down the cost of the procedure. Awareness programs, especially through seminars and workshops, need to be initiated - catering to both the general population and medical professionals. Importantly, these awareness programs need to be all-rounded, educating the people about bone health, the importance of timely DEXA scanning as well as recommendations for management and therapy, in case of disappointing results of the scans. Lastly, it would help to conduct similar awareness surveys especially targeted at the male population and youth in the third-world countries, as these are the people responsible for educating the women in their houses who have limited outreach to such services themselves.
